# Evolving Indications of Transcatheter Aortic Valve Replacement Compared to Surgical Valve Replacement: A Review of the Current Literature

**DOI:** 10.7759/cureus.23364

**Published:** 2022-03-21

**Authors:** Shitij Shrivastava, Shashwat Shrivastava, Kahkashan Mumtaz, Jihan A Mostafa

**Affiliations:** 1 Internal Medicine, California Institute of Behavioral Neurosciences & Psychology, Fairfield, USA; 2 Vascular Surgery, California Institute of Behavioral Neurosciences & Psychology, Fairfield, USA; 3 Medicine, California Institute of Behavioral Neurosciences & Psychology, Fairfield, USA; 4 Research and Publication, California Institute of Behavioral Neurosciences & Psychology, Fairfield, USA

**Keywords:** sts score, interventional cardiology corner, permanent pacemaker implantation (ppm), transcutaneous aortic valve replacement, minimally invazive valve surgery, structural valve degeneration, aortic stenosis (as), surgical aortic valve replacement (savr), transcatheter aortic valve implant, viv-tavr

## Abstract

Patients with severe symptomatic aortic stenosis (AS) are categorized into high risk, intermediate risk, and low risk. The identification of risk status is done using the Society of Thoracic Surgeons mortality score. Various factors are considered such as clinical symptoms, ejection fraction, age, left ventricle measurements, severity of AS, associated comorbid factors, and any other associated cardiac diseases. Surgery is still a standard practice in many countries. However, it has its own complications, especially in high-risk patients. Transcatheter intervention is getting precipitous recognition as an alternative mode of treatment in selected cases to mitigate complication rates and improve quality of life. In this article, transcatheter aortic valve replacement and surgical aortic valve replacement are compared in patients with different surgical risks. The impact of the cost of the procedure and quality of life are of paramount importance in choosing the type of intervention. Structural valve degeneration is an independent risk factor affecting patient outcomes. Modifications in valve designs are being constantly implemented as well. The standard analytical methods are in accordance with randomized clinical trials to determine the efficacy and outcome of procedures. Primary and secondary endpoints were considered to evaluate the data. The results were tabulated to derive statistical significance of the studies. In high-risk surgical patients, transcatheter intervention has been proven as the procedure of choice for valve replacement. However, intermediate-risk and low-risk categories need further studies.

## Introduction and background

The aortic valve is a tricuspid valve that serves as a connection between the left ventricle outflow tract and the aorta. It allows blood to flow from the left ventricle to the systemic circulation. The thin leaflets of the aortic valve can tolerate higher mechanical and hemodynamic pressures with each cardiac cycle due to the very complex and organized biological processes [1]. A range of factors, such as valvular degenerative changes and congenital anomalies such as bicuspid valve can impair normal valve function, resulting in a diverse range of clinical problems. In the industrialized world, aortic stenosis (AS) is the most common valvular heart disease where degenerative AS affects about 7% of the population over the age of 65 [2]. Recent meta-analyses show that the prevalence of AS in patients aged 60-64 years and more than 75 is 2.8% and 13.1%, respectively. This equates to an estimated 16.1 million people with AS. Among them, 1.0 million patients are suitable for transcatheter aortic valve replacement (TAVR) and 1.9 million patients are eligible for surgical aortic valve replacement (SAVR) [3]. In 1965, Davies first described a cone-shaped valve mounted over a catheter that was intended to reduce aortic regurgitation (AR) and this was tested in animals [4]. Later, in 2002, Dr. Alan Cribier, a French interventional cardiologist performed the first percutaneous heart valve implantation in a 57-year-old man who had contraindications for SAVR. A balloon aortic valvuloplasty had already been performed with nonsustained results. A remarkable functional improvement was observed in this patient and he could ease back into his life. The patient died 17 weeks later from noncardiac complications [5]. This milestone gathered further attention from various researchers and pharmaceutical companies and led to TAVR becoming a standard of care and having its indications expanded. Henning Rud Andersen, a Danish cardiologist, made the first animal implantation of what is now known as a transcatheter aortic valve replacement, in 1989, which resulted in a patent application filed in 1990 and approved in 1995 [6]. In recent years, a lot of studies have emerged comparing TAVR to SAVR and indications continue to rise. TAVR now encompasses 12.5% of all aortic valve procedures [7]. The awareness and prevalence of this procedure have increased dramatically ever since it was first approved for commercial use in 2007 in Europe and 2011 in the United States.

SAVR, TAVR or even medical treatment is determined by many factors, including concomitant diseases, surgical risk, and patient preference [8]. The patient should be explained in detail the risk involved, advantages of the procedure, and alternative modalities, if any. The interventionist’s experience in performing transcatheter-based procedures as well as the financial component are aspects of extreme importance [8]. Such physicians are trained in either interventional cardiology or cardiothoracic surgery. The cardiac catheterization laboratory should consist of well-trained nurses, anesthesiologists and must have a cardiac surgeon on standby.

**Figure 1:**
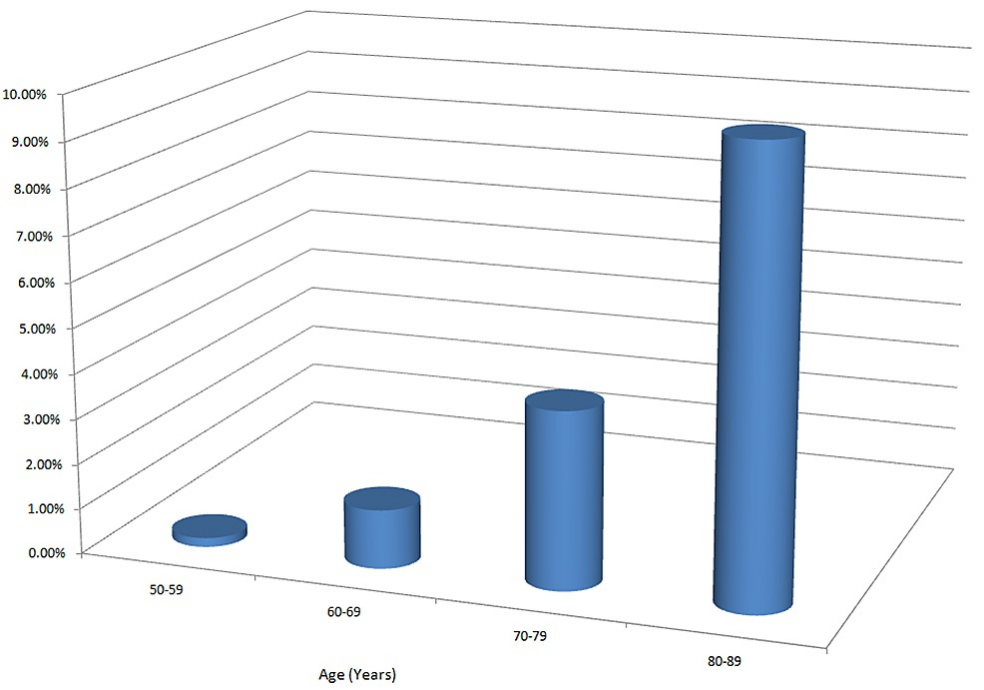
Prevalence of aortic valve stenosis by age

Here, we are going to discuss TAVR and SAVR with a review of the current literature and the evolution of TAVR from its use in high-risk to intermediate-risk and eventually low-risk surgical patients. How far has TAVR gone since it was first performed in 2002 with various researches that have taken place to expand its indications for use in the modern day cardiac catherization lab? A bar graph depicting the prevalence of aortic stenosis in different age groups is shown in Figure 1 [9].

## Review

Surgery has always been the gold standard in the treatment of severe AS with good results. However, TAVR has traveled a long way since its inception in 2002 and the indications have increased over the years [5]. The long-term effectiveness of the TAVR, however, is still under trial.

According to the 2020 American College of Cardiology/American Heart Association guidelines, there are well-defined indications for TAVR that address age, comorbidity, life expectancy, coronary anatomy, redo surgery, valve-in-valve replacement, and ejection fraction. Currently, TAVR is recommended in patients older than 80 who have a life expectancy of more than 10 years, irrespective of surgical risk, and in patients older than 80 years at high surgical risk with a life expectancy of at least one year. In symptomatic patients aged between 65 and 80, and asymptomatic patients <80 years old (ejection fraction <50%), either SAVR or TAVR can be performed [10]. The crucial question arises as to the long-term durability of the implanted valve. Also, patient-prosthetic mismatch is a vital issue. There are certain relative contraindications of a surgical valve replacement such as a porcelain aorta, redo surgery with patent coronary artery bypass grafts, and a history of excessive chest radiation where TAVR can be a suitable option [11].

TAVR versus SAVR in high-risk surgical patients

In the 2011 study, Smith et al. compared the transcatheter and surgical aortic valve replacement in patients with high surgical risk [12]. They randomly assigned 699 high-risk patients with aortic stenosis at 25 centers to undergo either TAVR or SAVR. Any-cause mortality at one year was the primary end point and they hypothesized that transcatheter replacement is noninferior to surgical replacement. Death rate was not significantly different (p = 0.07) between the two groups at 30 days. At one year, death rates for the transcatheter and surgical groups were 24.2% and 26.8%, respectively (p = 0.44) [12]. In 2016, Siontis et al. by a meta-analysis of randomized trials compared the safety and efficacy of transcatheter intervention and SAVR in severe aortic stenosis. A total of 1898 patients were assigned for TAVR and 1908 for SAVR from a total of 3806 cases. Here, the primary end point was all-cause mortality at two years. Transcatheter aortic valve implantation (TAVI), as opposed to SAVR, was associated with a 13% relative risk reduction (p = 0.038) in the primary outcome. TAVR demonstrated fewer secondary complications such as kidney injury, new-onset atrial fibrillation, and bleeding whereas SAVR showed significantly fewer vascular complications, requirement of a permanent pacemaker implantation (PPI), and paravalvular regurgitation [13].

In 2016, Deeb et al. evaluated three-year outcomes in high-risk patients. They assessed the sustainability of clinical gain over this prolonged period of time. A team identified 797 high-risk surgical patients who were randomized in equal proportions for TAVR and SAVR. Factors for high-risk identification were established as less than 50% major morbidity or surgical mortality at one month and 15% or higher risk of death at 30 days. Death or stroke at two years was taken as the primary end point [14].

TAVR fared significantly better than SAVR in terms of stroke frequency (12.6% vs. 19.0%, p = 0.034) at three years, and all-cause death or stroke rate combined at three years also revealed statistically significant results in favor of TAVR (TAVR, 37.3%, and surgery, 46.7%, p = 0.006). Echocardiographic parameters at three years disclosed better valve hemodynamics in the TAVR subgroup (mean gradient 7.62 ± 3.57 vs. 11.40 ± 6.81 mm Hg; p < 0.001). At three years, moderate to severe AR was found to be more prevalent in TAVR patients than surgical patients (6.8% vs. 0.0%; p < 0.001) though. Also, adverse cardiac and vascular neurologic events were lower when percutaneous implantation was done in contrast to SAVR (40.2% vs. 47.9%; p = 0.025). In surgery, the incidence of acute kidney injury (AKI) and life-threatening bleeding was higher but PPI, vascular problems, and reinterventions were greater in the TAVR group especially in the 30 days post-procedure. The incidence of structural valve degeneration (SVD) was also comparable in both groups. In SAVR, the surgeon chose the size and type of valve based on valve measurements during surgery. Also, the incidence of valve endocarditis, readmission, and clinical symptoms was similar in both groups at three years. The data revealed the three-year clinical benefit of TAVR over SAVR in high-risk groups [14]. Undoubtedly, TAVR has been an established option for high-risk aortic valve replacement for more than a decade.

TAVR versus SAVR in intermediate-risk surgical patients

With the increasing use of TAVR in high-risk patients, the indication was expanded for the intermediate risk groups as well by the FDA in 2016. Leon et al. with Placement of AoRTic TraNscathetER Valves (PARTNER) II investigators published their study on intermediate-risk groups (mean Society of Thoracic Surgeons [STS] score 5.8%). They conducted a multicentric randomized trial involving 57 centers including 2032 patients with severe AS and assigned them for either TAVR or SAVR. The primary end point was death from any cause or disabling stroke at two years. Out of those 2032 patients, 1021 were randomly assigned to SAVR and 1011 to TAVR. At two years, no significant difference could be witnessed in terms of the primary end point although TAVR with a transfemoral approach resulted in significantly lower death and stroke rates as compared to SAVR (p = 0.05). At two years, the all-cause mortality was 16.7% for TAVR and 18.0% for SAVR, and the disabling stroke rate was 6.2% for TAVR and 6.4% for SAVR. Except for vascular complications (7.9% vs. 5.0%, p = 0.008), most other complications such as life-threatening bleeding (10.4% vs. 43.4%, p < 0.001), AKI (1.3% vs. 3.1%, p = 0.006), and atrial fibrillation (9.1% vs. 26.4%, p < 0.001) were lower in the TAVR than the SAVR group. The results were similar in both groups given the primary end point. Both resulted in an equal degree of improvement in cardiac symptoms and readmission rates over the two years of trial [15].

Reardon et al. in 2017 presented data on TAVR in a group of 1746 intermediate-risk patients [16]. Of these patients who were randomized at 87 centers, only 1660 underwent surgical or transcatheter intervention. The Surgical Replacement and Transcatheter Aortic Valve Implantation (SURTAVI) trial was conducted to understand, analyse and compare the outcome of TAVR and surgery in intermediate-risk patients with severe AS. Bayesian analytical methods were applied to gauge the noninferiority of TAVR compared to surgery using a margin of 0.07. The mean STS score was 4.5% ± 1.6%. Here, the definition of intermediate surgical risk was an estimated risk of 30-day surgical death of 3%-15%. The primary end point was all-cause mortality or disabling stroke at 24 months, similar to the PARTNER II trial. The rate of death from any cause or disabling stroke was 12.6% in the TAVR group and 14% in the surgery group, which was consistent with noninferiority of TAVR (posterior probability of noninferiority, >0.999). All-cause mortality at 24 months in the TAVR group was 11.4% and 11.6% in the SAVR group. In both groups, a similar disabling stroke rate was observed. TAVR resulted in higher paravalvular leaks and pacemaker implantations whereas surgery was associated with increased blood loss, AKI, and new-onset atrial fibrillation [16].

Echocardiographic follow-up determined better aortic valve hemodynamics in TAVR with regard to the valve area and mean gradients. However, at one year, moderate to severe aortic regurgitation (AR) was 5.3% in the TAVR subset and 0.6% in surgery [16]. Compared to the PARTNER IIA trial, this trial had a lower mean STS score (5.8% vs. 4.5%). The SURTAVI trial had the lowest observed to expected 30-day surgical mortality ratio (0.38 vs. 0.71 in PARTNER IIA ) when the study results were published in 2017 [13,17]. They concluded that in intermediate-risk patients as classified by the multidisciplinary team, TAVR is noninferior to surgery statistically in terms of the primary end point at 24 months [16].

TAVR versus SAVR in low-risk surgical patients

Recently, studies are coming forth for the application of TAVR in low-risk patients and many trials are still being conducted. Braghiroli et al. reviewed PARTNER 3 and Evolut trials on the use of TAVR in low-risk patients [18]. The Evolut Low Risk trial randomized 1468 patients with severe aortic stenosis for TAVR and SAVR (mean STS 1.9%) whereas PARTNER 3 trial randomized 1000 patients (mean STS 1.9%). The incidence of death or disabling stroke at two years was 5.3% with TAVR and 6.7% with SAVR in the Evolut Low Risk trial. The PARTNER 3 trial observed its end point of all-cause death, stroke, or rehospitalization at one year to be 8.5% and 15.1% in TAVR and SAVR cases respectively. Thirty-day and one-year mortality in PARTNER 3 trial was 0.4% versus 1.1% and 1.0% versus 2.5% in TAVR and SAVR arms, respectively. In the Evolut Low Risk trial, 30-day mortality was 0.5% versus 1.3% for TAVR and SAVR, respectively [18].

In the PARTNER 3 trial, the outcome difference met the pre-determined criteria for noninferiority and superiority (p < 0.001 for TAVR and SAVR) whereas in the Evolut Low Risk trial, the difference met the noninferiority, but did not meet the superiority criteria. Both the groups had similar all-cause mortality and lower rates of other complications. However, the incidence of paravalvular leak and PPI was higher in TAVR subjects. Both the trials reported considerable improvements in the New York Heart Association (NYHA) class at 30 days and at one year irrespective of the type of procedure [18].

PARTNER 3 trial, 2021

Leon et al. studied outcomes at the two-year follow-up after TAVR in low-risk patients in 2021 [19]. In this study, both clinical and echocardiographic developments were determined at one and two years. The primary end point was death, stroke, or rehospitalization at one year. A total of 96.5% of patients were followed up for the primary end point at two years. This was lower in the TAVR subset (11.5%) as opposed to the SAVR subset (17.4%) with a p value of 0.007. However, death rate (p = 0.47) and stroke (p = 0.28) at two years were found not to be statistically significant. At two years, the death rate was 2.4% for the TAVR and 3.2% for the SAVR group; the stroke rate was 2.4% for TAVR and 3.6% for SAVR. Similar outcomes were observed on echocardiographic readings in terms of valve hemodynamics and structural degeneration at two years.

Cost effectiveness and quality of life

In Cohort A subset of the PARTNER trial, the researchers documented 12-month costs and quality-adjusted life years (QALYs). Transfemoral TAVR catalyzed cost mitigation of around $1250 less than surgery. Incremental cost effectiveness ratio (ICER) came down to $50,000/QALY gained in 70.9% of patients [20]. The Kansas City Cardiomyopathy Questionnaire summary scores for quality of life evaluation for transfemoral TAVR achieved higher values in the short term than the surgery cohort [21]. In CoreValve U.S. High Risk Pivotal Trial, the cost of SAVR in the intermediate-risk group was lower than anticipated and the lifelong cost expected was $17,849 that supported surgery. So the cost of TAVR versus SAVR was $55,090 per QALY achieved, depicting similar numbers calculated for TAVR versus medical management in the PARTNER Cohort B trial [13,22].

Valve durability

Blackman et al. studied long-term valve function and hemodynamic SVD after TAVR as defined by the European Task Force committee guidelines [23]. This study revealed no increase in mild AR (p = 0.02) and increased trivial AR (p = 0.002) compared with baseline imaging at a median follow-up of 5.8 years. Severe SVD was noticed in less than 0.5% of patients and moderate SVD was noted in 0.08% of patients. Addressing long- term valve durability in young and low-risk patients is a hot topic as it is crucial in expanding the indications for TAVR to low- and intermediate-risk patients.

Valve-in-TAVR

Raschpichler et al. in 2020 studied valve-in-TAVR (ViTAVR) for degenerated valves in patients who underwent either TAVR or surgery previously [24]. They compared the results of ViTAVR and valve-in-SAVR (ViSAVR). Amongst 99 patients in the study, 74.7% were ViSAVR cases and 25.3% of patients went through ViTAVR. The median STS score was 7.2%. They discovered significantly better valve gradients for ViTAVR (mean gradient 12.5 vs. 16.0 mm Hg in ViTAVR vs. ViSAVR; p = 0.045). Device success rates, mortality, and the need for PPI were found to be similar in both groups. Hence, ViTAVR can be a suitable option in patients at high or even intermediate risk when compared to surgery [24].

## Conclusions

In high-risk surgical patients, TAVR has been established as the first-line treatment option for severe aortic stenosis. In patients at intermediate risk, TAVR has been shown to be a viable alternative to SAVR. A heart team discussion must always take place prior to any TAVR procedure. Every procedure must be decided on a case-to-case basis and cardiac surgery should be consulted if adverse post-TAVR events occur. In low-risk young patients, the choice of TAVR is increasing, but more studies are needed. Further research must also be done on TAVR via transapical access. SURTAVI and PARTNER II trials have demonstrated encouraging results in intermediate-risk patients as well. Studies accessing the long-term valve durability are not yet available. Efforts should be directed towards mitigating the incidence of SVD. The causes of SVD are not well defined though it is considered to be multifactorial. Better engineering of valves and refinement in technology can play a role in curtailing causes of degeneration. The indications for TAVR have expanded in recent years, but SVD and paravalvular leaks need to be addressed in order to include young patients and bicuspid valves. TAVR is approved in high-risk patients, but SAVR is the gold standard of treatment in low-risk and young patients.
